# Supplementary material for: Neofunctionalization of an ancient domain allows parasites to avoid intraspecific competition by manipulating host behaviour
Source: Nat Commun. 2021 Sep 16;12:5489. doi: 10.1038/s41467-021-25727-9 (PMC8446075; doi:10.1038/s41467-021-25727-9)
Supplement: Supplementary file 1 — Supplementary Information [file 41467_2021_25727_MOESM1_ESM.pdf]

Supplementary Information for

**Neofunctionalization of an ancient domain allows parasites to avoid intraspecific competition by manipulating host behaviour**

Supplementary Figure 1 ------------------------------------- 2
Supplementary Figure 2 ------------------------------------- 3
Supplementary Figure 3 ------------------------------------- 4
Supplementary Figure 4 ------------------------------------- 5
Supplementary Figure 5 ------------------------------------- 6
Supplementary Figure 6 ------------------------------------- 7
Supplementary Figure 7 ------------------------------------- 8
Supplementary Figure 8 ------------------------------------- 9
Supplementary Figure 9 ------------------------------------ 10
Supplementary Figure 10 ------------------------------------ 11
Supplementary Figure 11 ------------------------------------ 12
Supplementary Table 1 -------------------------------------- 13
Supplementary Table 2 -------------------------------------- 14
Supplementary Table 3 -------------------------------------- 15
Supplementary Table 4 -------------------------------------- 16
Supplementary Table 5 -------------------------------------- 17

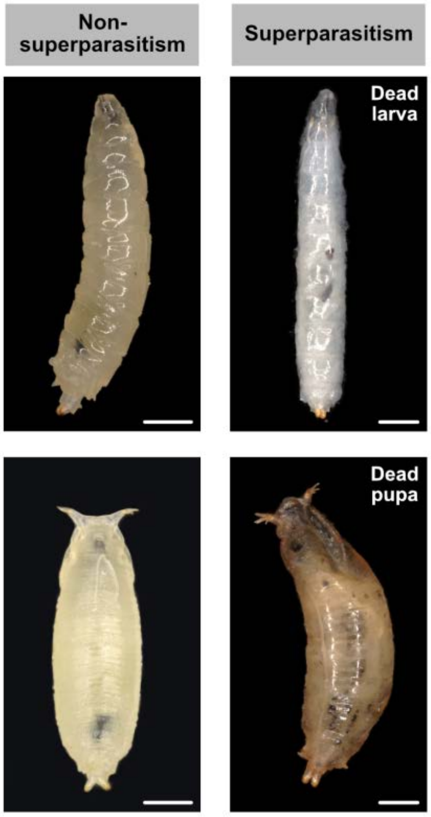


**Supplementary Figure 1 Representative images of premature death in superparasitized hosts**

The development of escaped (superparasitism avoidance) and non-escaped hosts (superparasitism) was observed in the 4 h parasitization assay. Bar: 1 mm. The photos were taken by Jia'ni Chen using a KEYENCE VHX-2000C digital microscope system.

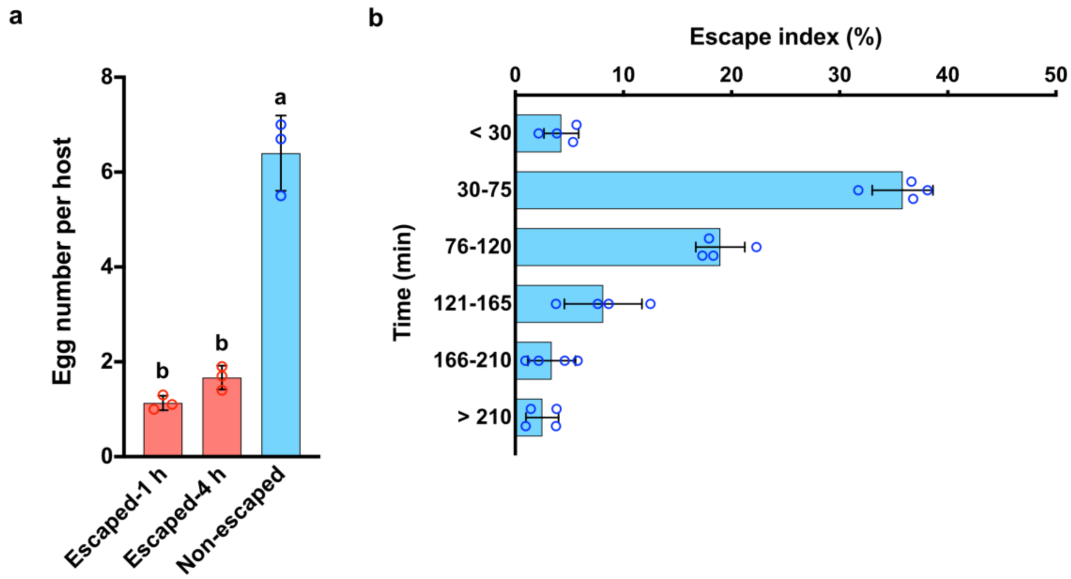


**Supplementary Figure 2 Host escape statistics across different time points after exposure to Lb parasitoids**

**a** Average number of parasitoid eggs found in escaped (red column, n=30) and non-escaped (blue column, n=30) hosts of *Drosophila* larvae after exposure to Lb at 1:10. The hosts were randomly selected from non-escaped and escaped groups after 4h exposure to Lb to check parasitoid eggs. The escaped hosts were additionally assayed at an early time point (1 h), which is the peak time of escape (see **b**). Three biological replicates were performed for each assay. Data are presented as mean values ± SD. Significance was analyzed by one-way ANOVA along with Fisher's least significant difference test (Escaped-1 h vs Escaped-4 h: $P$=0.4279; Escaped-1 h vs Non-escaped: $P=2.9\times10^{-5}$; Escaped-4 h vs Non-escaped: $P=5.4\times10^{-5}$). The differences between treatments were considered significant when $P<0.05$. **b** Escape preference of hosts across different time points after exposure to Lb at 1:10. Four biological replicates were performed for each assay. Data are presented as mean values ± SD. Source data are provided as a Source data file.

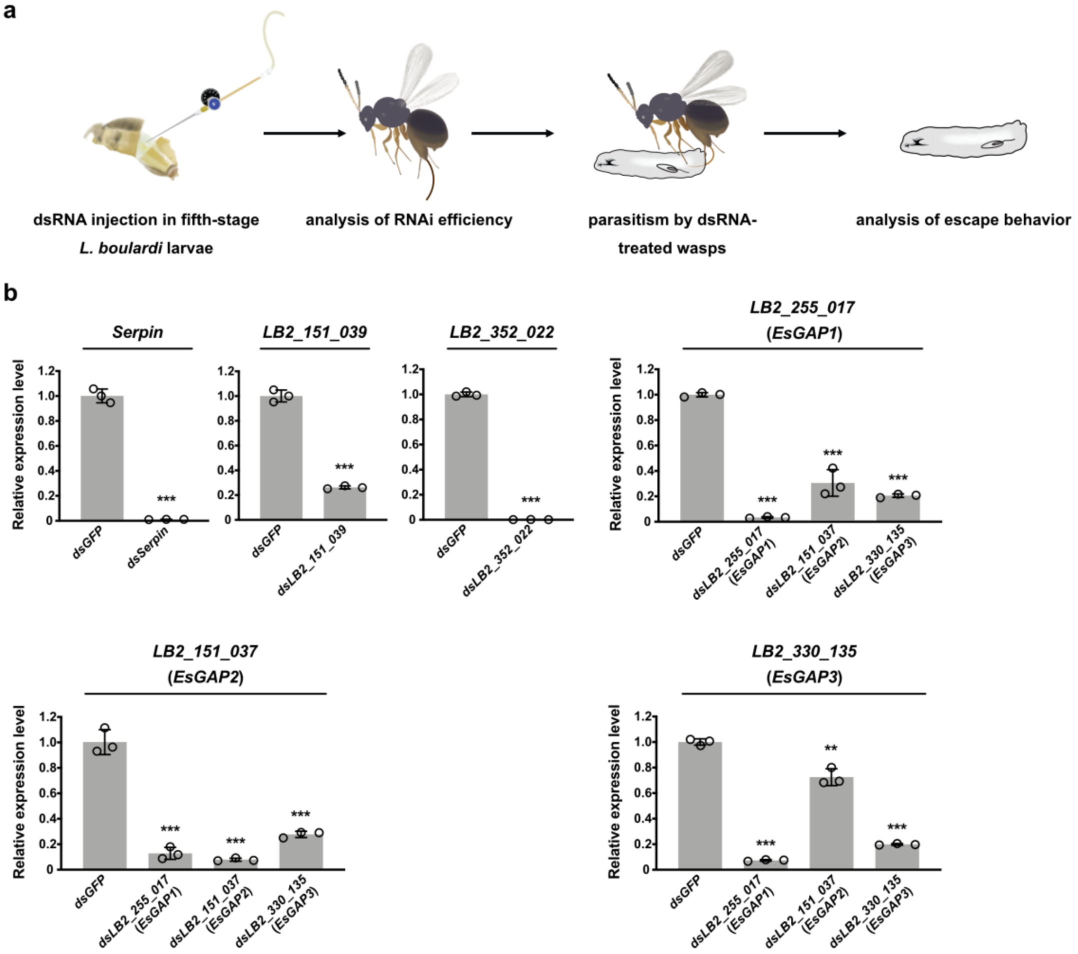


**Supplementary Figure 3 RNAi effects of significantly expressed VP genes on reducing gene expression**

**a** Schematic diagram of analyzing the role of candidate genes in host escape behaviour by inhibiting gene expression. The images were crafted using Affinity Designer v1.8.6 and Procreate v4.2.2. **b** Relative mRNA levels of all significantly highly expressed VP genes, including *Serpin*, *LB2_151_039*, *LB2_352_022*, *LB2_255_017* (*EsGAP1*), *LB2_151_037* (*EsGAP2*), and *LB2_330_135* (*EsGAP3*), in Lb venom apparatus after RNAi treatments. *dsGFP* was used as control. Three biological replicates were performed. Data are presented as mean values ± SD; significance was determined by two-sided unpaired Student's t-test (*dsSerpin*: $P=6.5\times10^{-6}$; *dsLB2_151_039*: $P=1.4\times10^{-5}$; *dsLB2_352_022*: $P=7.7\times10^{-8}$; *dsEsGAP1*: $P=8.2\times10^{-8}$ for *EsGAP1*, $P=0.0003$ for *EsGAP2*, $P=3.9\times10^{-7}$ for *EsGAP3*; *dsEsGAP2*: $P=0.0002$ for *EsGAP1*, $P=8.4\times10^{-5}$ for *EsGAP2*, $P=0.0002$ for *EsGAP3*; *dsEsGAP3*: $P=3.8\times10^{-7}$ for *EsGAP1*, $P=0.0024$ for *EsGAP2*, $P=6.4\times10^{-7}$ for *EsGAP3*;******: $P < 0.01$; *******: $P < 0.001$). Source data are provided as a Source data file.

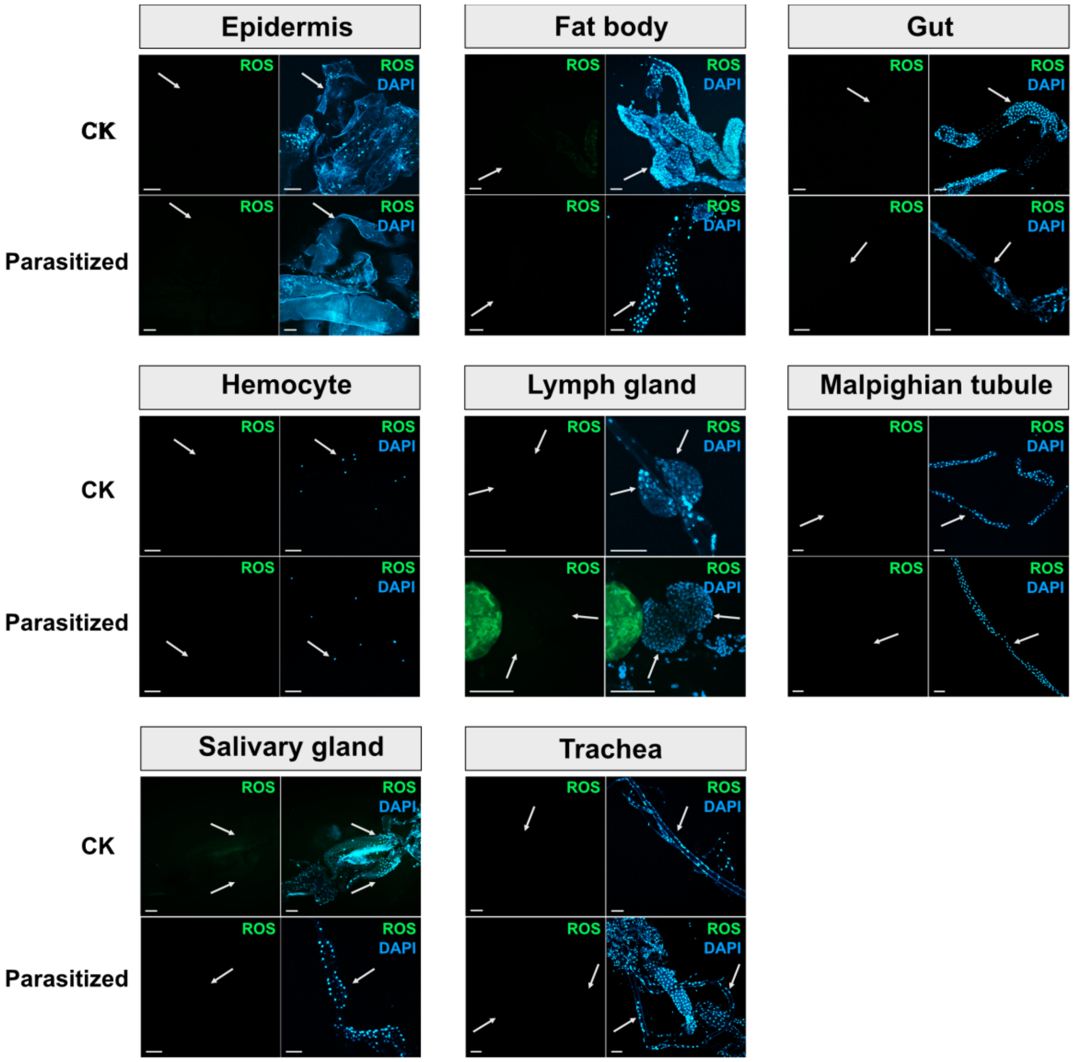


**Supplementary Figure 4 ROS levels in non-CNS host tissues after Lb parasitism**
Representative fluorescent images of epidermis, fat body, gut, hemocyte, lymph gland, malpighian tubule, salivary gland and trachea from a nonparasitized larva (CK) and from a parasitized host that exhibited escape behavior. Tissue was harvested immediately after 1 h post infection by Lb females. ROS levels were detected by DCFH-DA (green) and tissue nuclei were labeled by DAPI (blue). At least 20 *Drosophila* larvae were examined for each individual case. Arrows indicate the particular tissue. Scale bars: 50 μm.

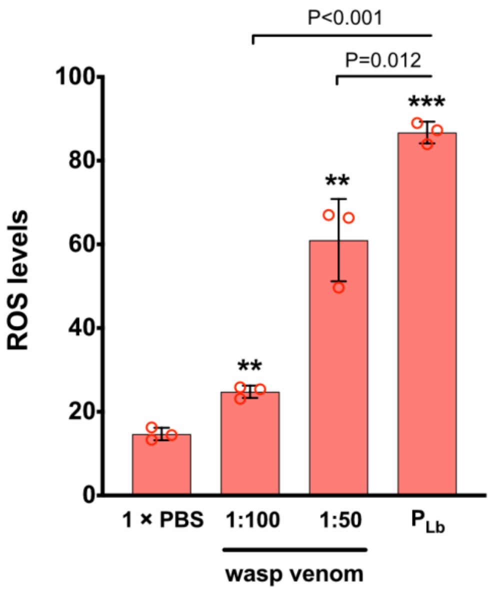


**Supplementary Figure 5 Lb venom triggers ROS induction in host CNS**
Quantification of ROS levels in CNS of larvae injected with different venom dilutions from a single Lb female wasp (n≥10 in each case). Elevation of ROS levels upon injection of 1:50 dilution of venom was much similar to the level in Lb parasitized host larvae (*P*=0.012). Data are presented as mean values ± SD. Significance was determined by two-sided unpaired Student's t-test (1:100 venom: *P*=0.0011; 1:50 venom: *P*=0.0013; $P_{Lb}$: *P*=$1.9x10^{-6}$; 1:100 venom vs. $P_{Lb}$: *P*=$3.5x10^{-6}$; 1:50 venom vs. $P_{Lb}$: *P*=0.012; **: *P* < 0.01; ***: *P* < 0.001). Source data are provided as a Source data file.

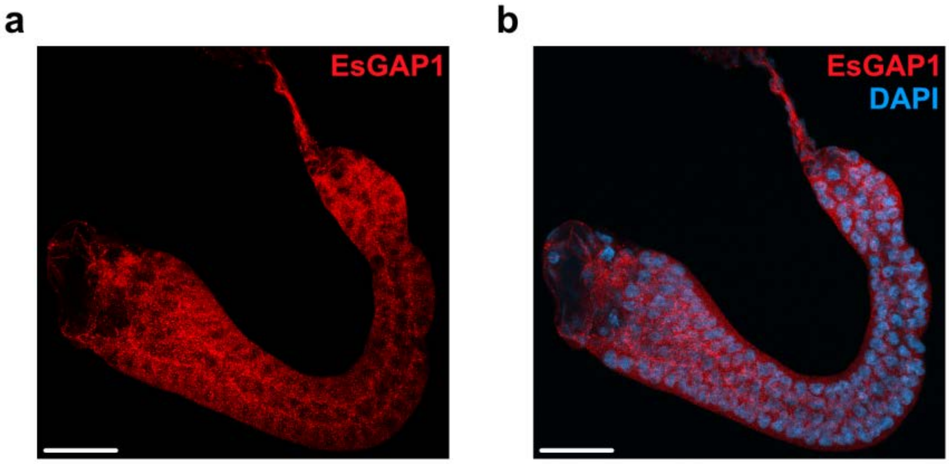


**Supplementary Figure 6 Immunolocalization assay of EsGAP1 expression in Lb**

**a** Confocal immunolocalization of EsGAP1 (red) in Lb venom gland. Venom glands of at least 20 parasitoids were examined. **b** Merged image of EsGAP1 staining (red) and nuclei stained with DAPI (blue). Scale bars: 50 μm.

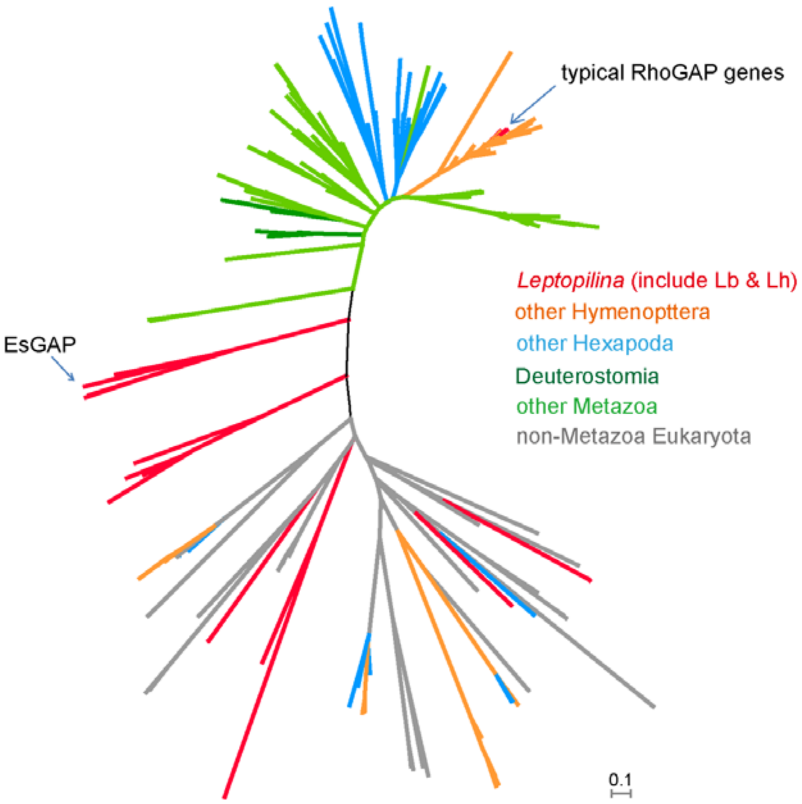


**Supplementary Figure 7 Phylogenetic analyses of RhoGAP genes across eukaryotes**

A total of 177 selective sequences from the representative nodes during animal evolution were used in this analysis (see Methods). Given the lower conservation out of the domain region, we retrieved the sequence of the RhoGAP domain (~170 AAs) from each full sequence prior to multiple alignment. The maximum-likelihood phylogenetic tree was inferred under the JTT model.

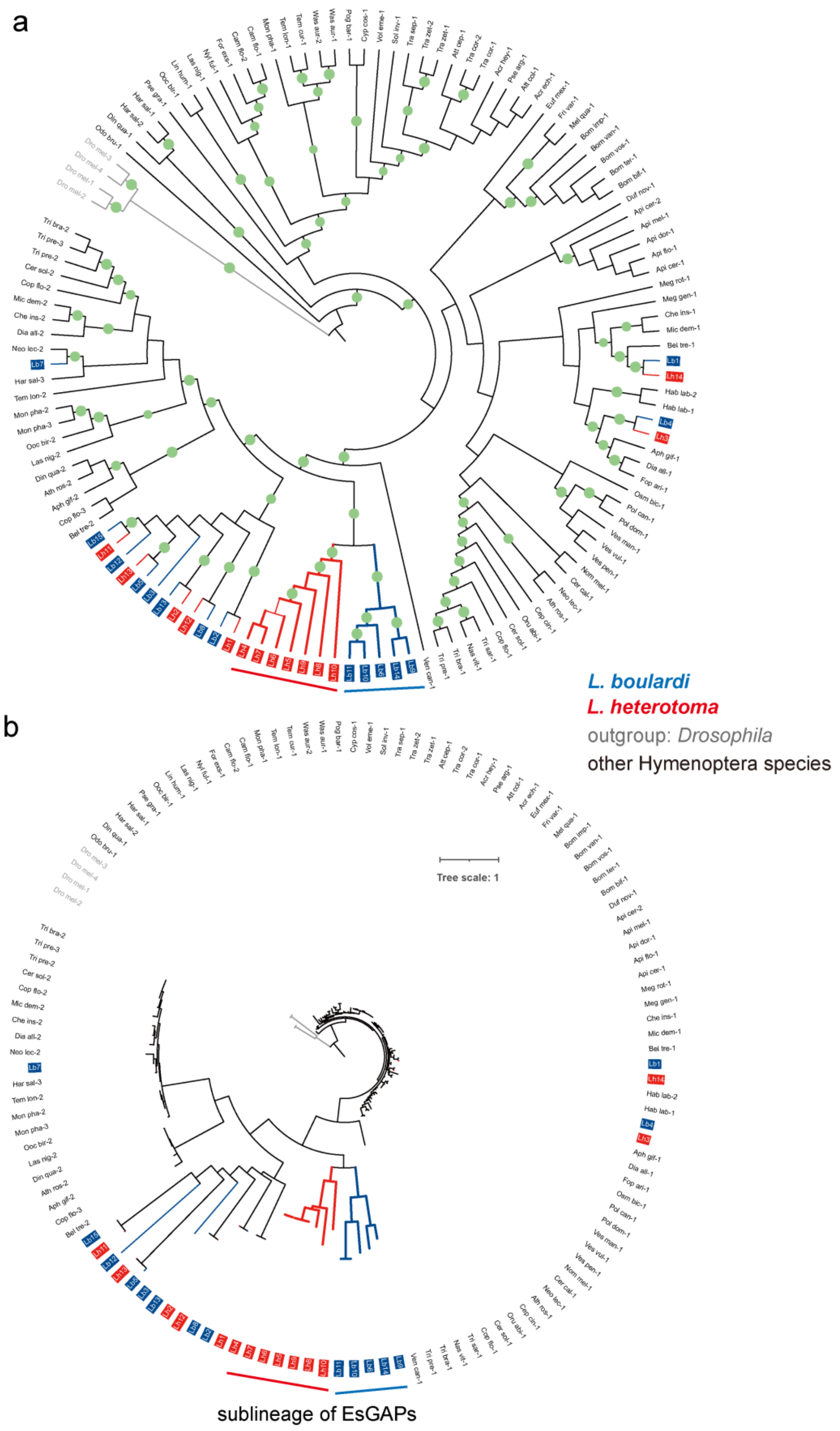


**Supplementary Figure 8 Phylogenetic analyses of RhoGAP genes across Hymenoptera** All 127 identified genes, from 70 hymenopteran species, were used in this analysis (see Methods). Only the sequences around the RhoGAP domain (165 AAs) from each full sequence were retained for analysis. The maximum-likelihood phylogenetic tree was inferred under the JTT model. **a** The tree in cladogram with labelled bootstraps. Nodes with green circles indicate being with >75% bootstrap supports. **b** The tree in phylogram with branch length. The sublineages of EsGAPs in Lb and Lh are underlined.

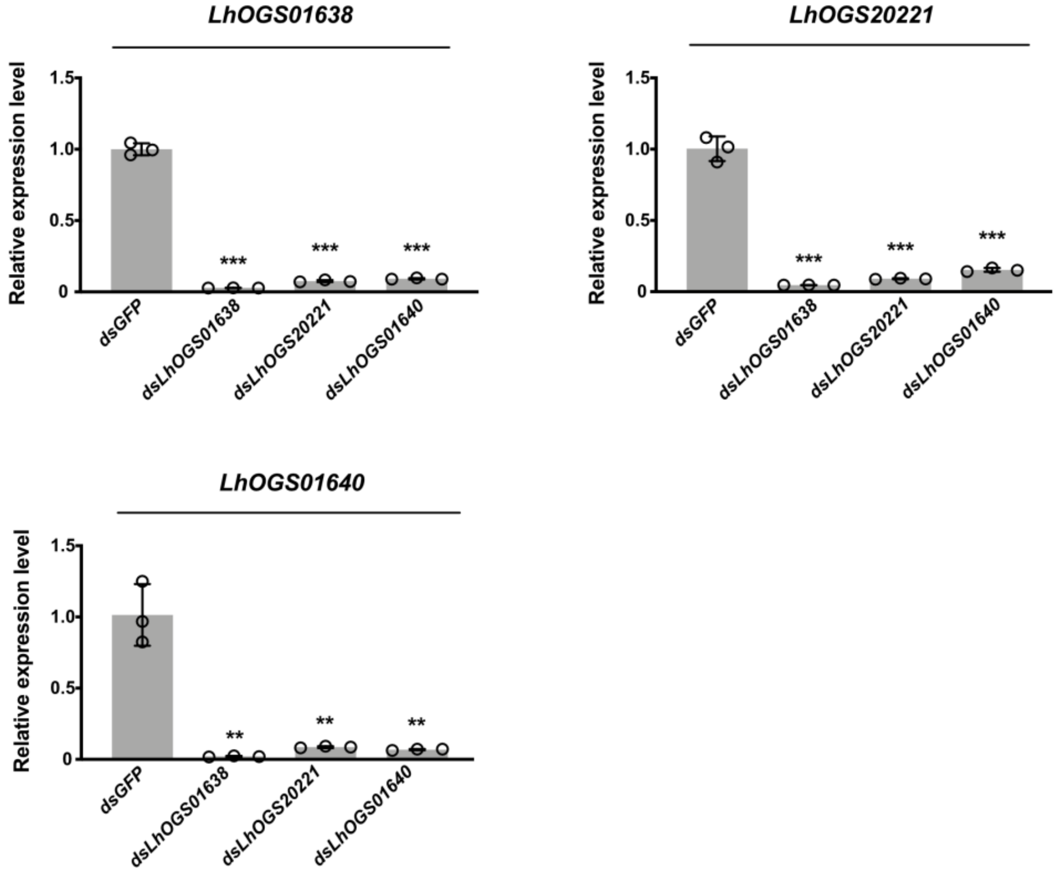


**Supplementary Figure 9 RNAi effects of three EsGAP genes on reducing gene expression in Lh**

Relative mRNA levels of *LhOGS01638* (LhEsGAP1), *LhOGS20221* (LhEsGAP2) and *LhOGS01640* (LhEsGAP3) in Lh venom apparatus after RNAi treatments. *dsGFP* was used as control. Three biological replicates were performed. Data are presented as mean values ± SD; significance was determined by two-sided unpaired Student's t-test (*dsLhOGS01638*: $P$=2.4x$10^{-6}$ for *dsLhOGS01638*, $P$=3.1x$10^{-6}$ for *dsLhOGS20221*, $P$=3.2x$10^{-6}$ for *dsLhOGS01640*; *dsLhOGS20221*: $P$=4.4x$10^{-5}$ for *dsLhOGS01638*, $P$=5.1x$10^{-5}$ for *dsLhOGS20221*, $P$=7.2x$10^{-5}$ for *dsLhOGS01640*; *dsLhOGS01640*: $P$=0.0014 *dsLhOGS01638*, $P$=0.0017 for *dsLhOGS20221*, $P$=0.0016 for *dsLhOGS01640*; **: $P < 0.01$; ***: $P < 0.001$). Source data are provided as a Source data file.

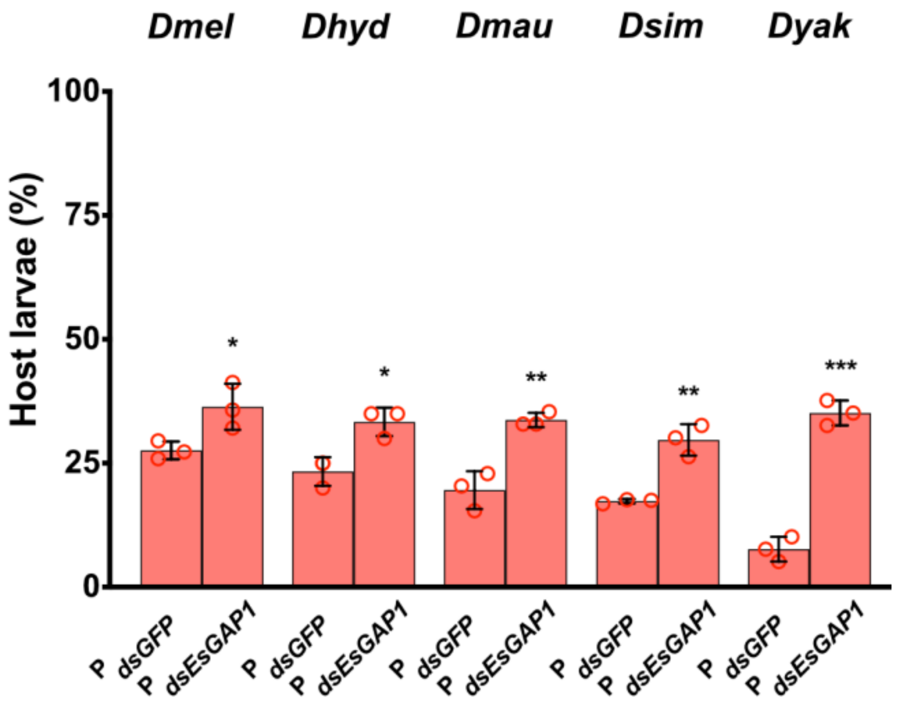


**Supplementary Figure 10 RNAi of EsGAP1 leads to more nonparasitized hosts remained under the condition of superparasitism**

Dissected hosts of without parasitoid eggs inside are considered as nonparasitized hosts. See the ratios of other fates (being parasitized only once and superparasitized) in Fig. 4a. Three biological replicates were performed. Data are presented as mean values ± SD. Significance was analysed by two-sided unpaired Student's t-test ($P_{EsGAP1}$ with *Dmel*: $P$=0.0376; $P_{EsGAP1}$ with *Dhyd*: $P$=0.0132; $P_{EsGAP1}$ with *Dmau*: $P$=0.0039; $P_{EsGAP1}$ with *Dsim*: $P$=0.0026; $P_{EsGAP1}$ with *Dyak*: $P$=0.0002; *: $P < 0.05$; **: $P < 0.01$; ***: $P < 0.001$). Dmel, *D. melanogaster*; Dhyd, *D. hydei*; Dmau, *D. mauritiana*; Dsim, *D. simulans*; Dyak, *D. yakuba*. Source data are provided as a Source data file.

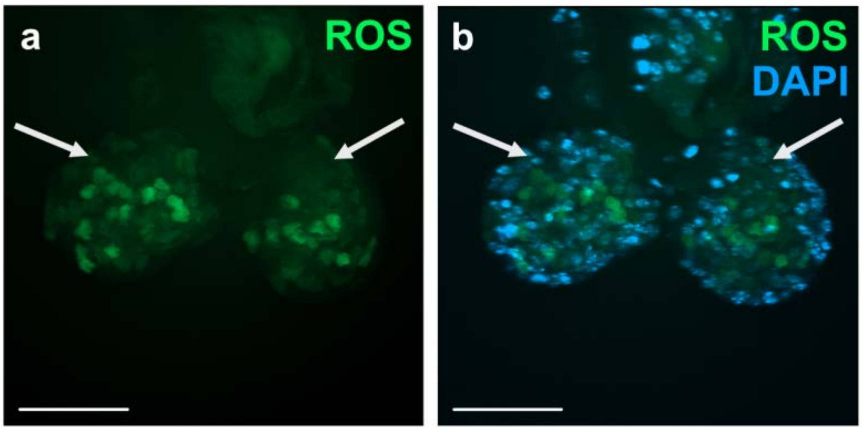


**Supplementary Figure 11 ROS levels in host lymph glands 12 hours after exposure to Lb**
**a** Representative fluorescent images of *Drosophila* lymph glands stained for ROS with DCFH-DA (green). **b** Merged image of tissue stained with DCFH-DA (green) and with DAPI (blue) to label nuclei. Larval lymph glands of at least 20 *Drosophila* were examined for each group. Lymph gland is indicated by arrows. Scale bars: 50 μm.

**Supplementary Table 1 Basic features of Lb genome assembly**

| | |
|---|---|
| Genome assembly | |
| Contig size (bp) | 354,804,354 |
| #Contigs | 409 |
| Contig $N_{50}$ (bp) | 2,668,382 |
| Genome annotation | |
| GC (%) | 28.0 |
| Repeats (simple repeats) (%) | 44.5 (3.6) |
| #Protein-coding genes | 12,613 |
| Coding (%) | 8.9 |
| Quality control | |
| BUSCO (insecta) partial (%) | 98.3 |
| BUSCO (insecta) complete (%) | 97.8 |
| BUSCO (hymenoptera) partial (%) | 95.4 |
| BUSCO (hymenoptera) complete (%) | 91.8 |
| CEGMA partial (%) | 97.2 |
| CEGMA complete (%) | 93.2 |

**Supplementary Table 2 Venom protein genes of Lb**

| Gene ID | #SP | TPM | Annotation* |
|---|---|---|---|
| LB2_216_018 | 25 | 100297 | Venom serpin |
| LB2_151_039s | 6 | 46364 | n/a |
| LB2_330_135 | 7 | 24835 | RhoGAP |
| LB2_352_022 | 5 | 23249 | n/a |
| LB2_255_017 | 19 | 16501 | RhoGAP |
| LB2_213_059 | 8 | 14031 | Superoxide dismutase |
| LB2_151_037 | 8 | 9248 | RhoGAP |
| LB2_248_134 | 17 | 9072 | Lipase |
| LB2_310_056s | 15 | 7161 | n/a |
| LB2_362_187s | 5 | 5641 | n/a |
| LB2_090_011 | 3 | 4134 | Calreticulin |
| LB2_273_020 | 8 | 3163 | Hypothetical protein |
| LB2_325_090 | 5 | 2915 | n/a |
| LB2_259_013s | 5 | 2859 | n/a |
| LB2_405_013 | 3 | 2794 | Hypothetical protein |
| LB2_223_093 | 20 | 2597 | Hypothetical protein |
| LB2_229_038 | 4 | 2438 | Elongation factor 1-alpha |
| LB2_055_066s | 20 | 1910 | Ubiquitin |
| LB2_064_044 | 15 | 1849 | Hypothetical protein |
| LB2_328_010 | 12 | 1558 | n/a |
| LB2_392_037 | 16 | 1524 | Hypothetical protein |
| LB2_269_001 | 8 | 1364 | Lysozyme |
| LB2_223_100 | 10 | 1317 | Hypothetical protein |
| LB2_032_074 | 5 | 1125 | Heat shock 70 kDa protein |
| LB2_368_216 | 5 | 996 | Peptidylprolyl isomerase |
| LB2_351_090 | 17 | 941 | Aminopeptidase |
| LB2_202_072 | 4 | 897 | ribosomal protein L40 |
| LB2_362_047 | 16 | 852 | Serine protease easter |
| LB2_195_052s | 64 | 761 | n/a |
| LB2_195_039 | 57 | 733 | n/a |
| LB2_169_090 | 39 | 710 | n/a |
| LB2_273_025s | 5 | 608 | n/a |
| LB2_351_102s | 16 | 562 | n/a |
| LB2_302_125 | 6 | 549 | Hypothetical protein |
| LB2_040_036 | 16 | 481 | Hypothetical protein |
| LB2_202_067 | 6 | 474 | Hypothetical protein |
| LB2_121_078 | 21 | 376 | Hypothetical protein |
| LB2_278_199 | 11 | 331 | Actin-5C |
| LB2_351_089 | 7 | 324 | Aminopeptidase |
| LB2_259_015 | 7 | 317 | n/a |
| LB2_249_032 | 8 | 316 | Hypothetical protein |
| LB2_399_119 | 25 | 303 | Hypothetical protein |
| LB2_377_061 | 3 | 297 | CALM_0 protein |
| LB2_202_100 | 3 | 272 | Hypothetical protein |
| LB2_310_078 | 3 | 268 | n/a |
| LB2_248_036 | 23 | 208 | n/a |
| LB2_302_012 | 11 | 203 | Hypothetical protein |
| LB2_258_003 | 12 | 192 | Mmel1_3 protein |
| LB2_112_062 | 6 | 189 | Trehalase |
| LB2_038_141 | 8 | 184 | Endoplasmic reticulum ATPase |
| LB2_210_047 | 8 | 183 | Leukotriene A(4) hydrolase |
| LB2_399_112 | 7 | 181 | Ionotropic glutamate receptor |
| LB2_300_065 | 7 | 152 | Hypothetical protein |
| LB2_038_115 | 4 | 152 | Heat shock |
| LB2_055_006s | 5 | 140 | n/a |
| LB2_080_098 | 3 | 130 | Nucleobindin-2 |
| LB2_318_034 | 17 | 112 | Lysosomal alpha-mannosidase |
| LB2_126_037 | 5 | 108 | Hypothetical protein |
| LB2_384_006 | 6 | 101 | Catalase |
| LB2_384_064 | 3 | 97 | Protein toll |
| LB2_342_106 | 3 | 95 | Hypothetical protein |
| LB2_123_060 | 7 | 90 | GTP-binding protein 2 |
| LB2_195_053s | 47 | 89 | n/a |
| LB2_202_122 | 3 | 88 | Importin subunit alpha |
| LB2_248_035 | 29 | 85 | n/a |
| LB2_032_029 | 3 | 52 | Cytochrome P450 6a2 |
| LB2_342_009 | 8 | 49 | Hypothetical protein |
| LB2_041_025s | 6 | 40 | n/a |
| LB2_362_044 | 3 | 40 | n/a |
| LB2_055_010 | 4 | 39 | n/a |
| LB2_384_060 | 4 | 34 | Hypothetical protein |
| LB2_055_008 | 4 | 30 | n/a |
| LB2_055_131 | 3 | 25 | CLIP-associating protein |
| LB2_165_094 | 3 | 23 | Oligomeric Golgi complex |
| LB2_055_040 | 4 | 22 | n/a |
| LB2_193_063 | 3 | 21 | Hypothetical protein |
| LB2_210_050 | 3 | 20 | Hypothetical protein |
| LB2_216_153 | 3 | 18 | Arginine kinase |
| LB2_362_055 | 5 | 17 | Transgelin |
| LB2_055_009 | 7 | 14 | n/a |
| LB2_316_055 | 3 | 14 | Hypothetical protein |
| LB2_055_007 | 3 | 13 | n/a |
| LB2_273_001 | 5 | 12 | Ankyrin-2 |
| LB2_310_087 | 3 | 12 | Spectrin beta chain |
| LB2_179_055 | 4 | 11 | Hypothetical protein |
| LB2_151_033 | 3 | 11 | Hypothetical protein |
| LB2_392_160 | 4 | 11 | retinitis pigmentosa GTPase |
| LB2_112_177 | 3 | 10 | Hypothetical protein |
| LB2_195_051 | 40 | 9 | n/a |
| LB2_123_202 | 3 | 8 | Hypothetical protein |
| LB2_112_123 | 3 | 8 | Hypothetical protein |

*SP, the count of peptide detected by proteome with 100% sequence identity; TPM, transcripts per million, normalized expression value of RNAseq; Annotation, the best BLASTP hit against the UniProt database. Genes in blue background indicate of significantly high expression level in the venoms (Z-test, *P*<0.01).

**Supplementary Table 3 Homologous regions encoding the RhoGAP domain in Lb and Lh genomes**

| Contig ID[a] | Strand | Locus position | Corresponding OGS ID[b] |
|---|---|---|---|
| LbouV2_1 | + | 868,856-872,281 | LB2_001_063 |
| Lhet_53 | + | 395,566-399,842 | LhOGS03178 |
| LbouV2_150 | - | 1,303,091-1,309,834 | LB2_150_055 |
| Lhet_18 | - | 1,465,913-1,480,789 | LhOGS01184 |
| LbouV2_169 | + | 2,154,376-2,164,465 | LB2_169_103 |
| LbouV2_278 | + | 4,265,093-4,278,742 | LB2_278_172 |
| Lhet_405 | + | 195,397-219,634 | LhOGS11401 |
| LbouV2_371 | + | 3,353,377-3,392,780 | LB2_371_076 |
| Lhet_108 | + | 2,221,126-2,327,245 | LhOGS06035 |
| LbouV2_1 | - | 1,587,155-1,587,806 | LB2_001_148 |
| LbouV2_179 | - | 957,879-979,728 | LB2_179_004 |
| Lhet_42 | - | 3,480,592-3,513,911 | LhOGS02298 |
| Lhet_166 | + | 1,359,807-1,364,726 | LhOGS07661 |
| LbouV2_302 | + | 1,597,777-1,610,241 | LB2_302_118 |
| LbouV2_112 | + | 3,034,506-3,043,583 | LB2_112_162 |
| LbouV2_151 | - | 64,210-70,326 | LB2_151_005 |
| Lhet_52 | + | 5,133,557-5,148,895 | LhOGS03040 |
| Lhet_26 | + | 4,080,189-4,080,907 | n/a |
| Lhet_26 | + | 863,738-8,644,42 | Lh-EsGAP3[c] |
| Lhet_26 | + | 845,813-852,290 | Lh-EsGAP2 |
| Lhet_26 | + | 741,969-742,519 | Lh-EsGAP1’[d] |
| Lhet_26 | + | 770,024-770,574 | Lh-EsGAP1’[d] |
| Lhet_26 | + | 759,013-759,563 | Lh-EsGAP1’[d] |
| Lhet_26 | + | 730,957-731,507 | Lh-EsGAP1 |
| LbouV2_216 | - | 4,232,052-4,235,053 | LB2_216_152 |
| LbouV2_330 | + | 2,748,394-2,753,571 | EsGAP3 |
| LbouV2_151 | - | 822,831-823,968 | EsGAP2[e] |
| LbouV2_255 | + | 620,159-620,919 | EsGAP1’[f] |
| LbouV2_255 | + | 601,067-601,827 | EsGAP1 |

The order of loci listed here corresponds to those shown in Fig. 5a (from top to bottom).
[a] Contig IDs from Lb (initiated with Lb) and Lh genomes (initiated with Lh), respectively; [b] the predicted OGS gene that encompasses the locus encoding the domain; [c] the length ratio of domain to the entire length of OGS; [d] this gene model concatenates four neighboring homologous loci, indicative of very recently duplication; [e] this gene is near-identical to a previously described LbGAP gene GU300066.1, but short by 39- and 126-bp of non-domain sequences on 5’- and 3’- ends, respectively; [f] this locus has no gene being predicted but shows high identity to its neighboring locus that encompassing LB2_255_017.

**Supplementary Table 4 Detailed statistics analysis of Fig. 1d**

| Time (min) | CK | Female wasps | Male wasps | *P* value | | |
|---|---|---|---|---|---|---|
| | | | | CK vs Female wasps | CK vs Male wasps | Female wasps vs Male wasps |
| 0 | 0.00 ± 0.00 | 0.00 ± 0.00 | 0.00 ± 0.00 | — | — | — |
| 15 | 0.00 ± 0.00 $^{b}$ | 1.91 ± 0.84 $^{a}$ | 0.00 ± 0.00 $^{b}$ | 0.0070 | 0.9999 | 0.0070 |
| 30 | 0.00 ± 0.00 $^{b}$ | 5.31 ± 2.08 $^{a}$ | 0.00 ± 0.00 $^{b}$ | 0.0040 | 0.9999 | 0.0040 |
| 45 | 0.00 ± 0.00 $^{b}$ | 20.36 ± 5.40 $^{a}$ | 0.61 ± 0.53 $^{b}$ | 0.0005 | 0.9689 | 0.0006 |
| 60 | 0.24 ± 0.41 $^{b}$ | 32.67 ± 6.30 $^{a}$ | 1.18 ± 0.67 $^{b}$ | 9.0e-5 | 0.9479 | 0.0001 |
| 75 | 0.24 ± 0.41 $^{b}$ | 42.41 ± 6.26 $^{a}$ | 2.03 ± 0.78 $^{b}$ | 1.8e-5 | 0.8248 | 2.4e-5 |
| 90 | 0.24 ± 0.41 $^{b}$ | 50.42 ± 7.07 $^{a}$ | 2.43 ± 0.87 $^{b}$ | 1.4e-5 | 0.7985 | 1.8e-5 |
| 105 | 0.52 ± 0.45 $^{b}$ | 56.80 ± 6.17 $^{a}$ | 2.43 ± 0.87 $^{b}$ | 3.0e-5 | 0.7992 | 3.7e-6 |
| 120 | 0.52 ± 0.45 $^{b}$ | 60.53 ± 7.01 $^{a}$ | 2.43 ± 0.87 $^{b}$ | 4.3e-6 | 0.8390 | 5.3e-6 |
| 135 | 0.75 ± 0.72 $^{b}$ | 63.53 ± 7.87 $^{a}$ | 2.95 ± 0.40 $^{b}$ | 6.5e-6 | 0.8299 | 8.1e-6 |
| 150 | 0.75 ± 0.72 $^{b}$ | 66.03 ± 7.40 $^{a}$ | 3.48 ± 0.09 $^{b}$ | 3.5e-6 | 0.7283 | 4.5e-6 |
| 165 | 1.03 ± 0.90 $^{b}$ | 69.46 ± 6.19 $^{a}$ | 3.64 ± 0.21 $^{b}$ | 1.2e-6 | 0.6691 | 1.4e-6 |
| 180 | 1.03 ± 0.90 $^{b}$ | 71.44 ± 5.30 $^{a}$ | 3.81 ± 0.49 $^{b}$ | 5.5e-7 | 0.5537 | 6.6e-7 |
| 195 | 1.55 ± 0.66 $^{b}$ | 73.94 ± 4.92 $^{a}$ | 3.97 ± 0.77 $^{b}$ | 3.4e-7 | 0.5907 | 4.0e-7 |
| 210 | 1.83 ± 0.88 $^{b}$ | 75.04 ± 5.03 $^{a}$ | 4.21 ± 0.69 $^{b}$ | 3.7e-7 | 0.6141 | 4.3e-7 |
| 225 | 1.83 ± 0.88 $^{b}$ | 76.45 ± 4.61 $^{a}$ | 4.21 ± 0.69 $^{b}$ | 2.0e-7 | 0.5670 | 2.5e-7 |
| 240 | 1.83 ± 0.88 $^{b}$ | 77.78 ± 5.04 $^{a}$ | 4.21 ± 0.69 $^{b}$ | 3.0e-7 | 0.6146 | 3.6e-7 |

One-way analysis of variance (ANOVA) along with Fisher's least significant difference tests was used. Data are presented as the mean ± standard deviation (mean ± SD) and differences between treatments are considered significant when $P < 0.05$. All statistical analyses were conducted using SPSS 26.0 Statistics and EXCEL.

**Supplementary Table 5 Detailed statistics analysis of Fig. 4c (*D. hydei*)**

| Time (min) | $P_{Lb}$ | CK | $P_{dsEsGAP1}$ | *P* value | | |
|---|---|---|---|---|---|---|
| | | | | $P_{Lb}$ vs CK | $P_{Lb}$ vs $P_{dsEsGAP1}$ | CK vs $P_{dsEsGAP1}$ |
| 0 | 0.00 ± 0.00 | 0.00 ± 0.00 | 0.00 ± 0.00 | — | — | — |
| 15 | 0.00 ± 0.00 | 0.00 ± 0.00 | 0.00 ± 0.00 | — | — | — |
| 30 | 3.98 ± 2.24 [a] | 0.00 ± 0.00 [b] | 1.00 ± 1.00 [b] | 0.0317 | 0.0923 | 0.6791 |
| 45 | 22.20 ± 12.66 [a] | 0.00 ± 0.00 [b] | 3.33 ± 1.15 [b] | 0.0234 | 0.0453 | 0.8472 |
| 60 | 37.43 ± 15.05 [a] | 0.24 ± 0.41 [b] | 6.00 ± 1.00 [b] | 0.0047 | 0.0106 | 0.7108 |
| 75 | 47.94 ± 12.64 [a] | 0.24 ± 0.41 [b] | 7.67 ± 1.53 [b] | 0.0005 | 0.0013 | 0.4763 |
| 90 | 55.56 ± 12.15 [a] | 0.24 ± 0.41 [b] | 9.67 ± 0.58 [b] | 0.0002 | 0.0005 | 0.2997 |
| 105 | 59.67 ± 11.43 [a] | 0.52 ± 0.45 [b] | 11.67 ± 2.08 [b] | 9.2e-5 | 0.0003 | 0.1845 |
| 120 | 66.59 ± 8.39 [a] | 0.52 ± 0.45 [c] | 13.00 ± 1.73 [b] | 7.8e-6 | 2.8e-5 | 0.0487 |
| 135 | 70.23 ± 7.83 [a] | 0.75 ± 0.72 [c] | 14.67 ± 2.08 [b] | 4.1e-6 | 1.6e-5 | 0.0256 |
| 150 | 72.79 ± 5.62 [a] | 0.75 ± 0.72 [c] | 15.67 ± 2.89 [b] | 1.0e-6 | 3.0e-6 | 0.0060 |
| 165 | 73.74 ± 4.78 [a] | 1.03 ± 0.90 [c] | 17.33 ± 1.53 [b] | 3.6e-7 | 1.1e-6 | 0.0012 |
| 180 | 75.30 ± 3.57 [a] | 1.03 ± 0.90 [c] | 18.00 ± 2.00 [b] | 8.6e-8 | 4.4e-7 | 0.0003 |
| 195 | 76.77 ± 2.14 [a] | 1.55 ± 0.66 [c] | 19.00 ± 2.00 [b] | 1.7e-9 | 4.3e-8 | 4.3e-5 |
| 210 | 77.25 ± 1.73 [a] | 1.83 ± 0.88 [c] | 20.00 ± 3.00 [b] | 1.6e-8 | 1.8e-7 | 9.3e-5 |
| 225 | 78.43 ± 2.21 [a] | 1.83 ± 0.88 [c] | 20.67 ± 2.52 [b] | 9.3e-9 | 1.4e-7 | 6.3e-5 |
| 240 | 78.43 ± 2.21 [a] | 1.83 ± 0.88 [c] | 20.67 ± 2.52 [b] | 9.3e-9 | 1.4e-7 | 6.3e-5 |

One-way analysis of variance (ANOVA) along with Fisher's least significant difference tests was used. Data are presented as the mean ± standard deviation (mean ± SD) and differences between treatments are considered significant when $P < 0.05$. All statistical analyses were conducted using SPSS 26.0 Statistics and EXCEL.

**Supplementary Table 5 Detailed statistics analysis of Fig. 4c (*D. mauritiana*)**

| Time (min) | $P_{Lb}$ | CK | $P_{dsEsGAP1}$ | *P* value | | |
|---|---|---|---|---|---|---|
| | | | | $P_{Lb}$ vs CK | $P_{Lb}$ vs $P_{dsEsGAP1}$ | CK vs $P_{dsEsGAP1}$ |
| 0 | 0.00 ± 0.00 | 0.00 ± 0.00 | 0.00 ± 0.00 | — | — | — |
| 15 | 3.13 ± 0.20 [a] | 0.00 ± 0.00 [b] | 0.00 ± 0.00 [b] | 1.8e-7 | 1.8e-7 | 0.9999 |
| 30 | 11.19 ± 3.14 [a] | 0.00 ± 0.00 [b] | 1.83 ± 0.76 [b] | 0.0008 | 0.0021 | 0.4932 |
| 45 | 19.06 ± 2.63 [a] | 0.55 ± 0.95 [b] | 3.92 ± 1.01 [b] | 2.9e-5 | 9.2e-5 | 0.1154 |
| 60 | 27.15 ± 3.37 [a] | 0.55 ± 0.95 [c] | 6.42 ± 1.51 [b] | 1.4e-5 | 6.3e-5 | 0.0391 |
| 75 | 34.87 ± 3.55 [a] | 0.55 ± 0.95 [c] | 8.75 ± 3.70 [b] | 2.1e-5 | 0.0001 | 0.0360 |
| 90 | 40.94 ± 3.02 [a] | 0.97 ± 0.86 [c] | 9.83 ± 3.62 [b] | 4.7e-6 | 2.2e-5 | 0.0182 |
| 105 | 46.05 ± 3.80 [a] | 0.97 ± 0.86 [c] | 10.83 ± 3.82 [b] | 5.0e-6 | 2.3e-5 | 0.0202 |
| 120 | 52.18 ± 3.96 [a] | 0.97 ± 0.86 [c] | 11.17 ± 3.75 [b] | 2.6e-6 | 9.9e-6 | 0.0185 |
| 135 | 56.85 ± 3.14 [a] | 0.97 ± 0.86 [c] | 11.83 ± 4.25 [b] | 1.4e-6 | 4.5e-6 | 0.0121 |
| 150 | 60.03 ± 3.66 [a] | 1.40 ± 1.30 [c] | 12.25 ± 3.63 [b] | 1.1e-6 | 3.0e-6 | 0.0117 |
| 165 | 62.74 ± 4.72 [a] | 1.40 ± 1.30 [c] | 12.58 ± 3.64 [b] | 1.7e-6 | 5.2e-6 | 0.0190 |
| 180 | 64.52 ± 5.72 [a] | 1.78 ± 0.72 [c] | 12.58 ± 3.64 [b] | 2.7e-6 | 8.4e-6 | 0.0351 |
| 195 | 66.01 ± 5.38 [a] | 1.78 ± 0.72 [c] | 13.33 ± 3.06 [b] | 1.5e-6 | 4.4e-6 | 0.0180 |
| 210 | 66.86 ± 5.87 [a] | 1.78 ± 0.72 [c] | 14.08 ± 2.50 [b] | 1.6e-6 | 5.2e-6 | 0.0156 |
| 225 | 67.18 ± 6.35 [a] | 1.78 ± 0.72 [c] | 14.08 ± 2.50 [b] | 2.2e-6 | 7.6e-6 | 0.0209 |
| 240 | 67.18 ± 6.35 [a] | 1.78 ± 0.72 [c] | 14.08 ± 2.50 [b] | 2.2e-6 | 7.6e-6 | 0.0209 |

One-way analysis of variance (ANOVA) along with Fisher's least significant difference tests was used. Data are presented as the mean ± standard deviation (mean ± SD) and differences between treatments are considered significant when $P < 0.05$. All statistical analyses were conducted using SPSS 26.0 Statistics and EXCEL.

**Supplementary Table 5 Detailed statistics analysis of Fig. 4c (*D. simulans*)**

| Time (min) | $P_{Lb}$ | CK | $P_{dsEsGAP1}$ | *P* value | | |
|---|---|---|---|---|---|---|
| | | | | $P_{Lb}$ vs CK | $P_{Lb}$ vs $P_{dsEsGAP1}$ | CK vs $P_{dsEsGAP1}$ |
| 0 | 0.00 ± 0.00 | 0.00 ± 0.00 | 0.00 ± 0.00 | — | — | — |
| 15 | 1.11 ± 1.92 [a] | 0.00 ± 0.00 [a] | 0.00 ± 0.00 [a] | 0.4827 | 0.4827 | 0.9999 |
| 30 | 10.70 ± 5.35 [a] | 0.00 ± 0.00 [b] | 2.92 ± 0.72 [b] | 0.0133 | 0.0504 | 0.5231 |
| 45 | 23.67 ± 8.88 [a] | 0.00 ± 0.00 [b] | 5.42 ± 1.91 [b] | 0.0035 | 0.0125 | 0.4622 |
| 60 | 33.16 ± 6.04 [a] | 0.41 ± 0.70 [c] | 8.75 ± 3.31 [b] | 0.0001 | 0.0007 | 0.0949 |
| 75 | 38.71 ± 6.89 [a] | 0.41 ± 0.70 [c] | 10.42 ± 4.39 [b] | 0.0002 | 0.0008 | 0.0912 |
| 90 | 43.23 ± 5.87 [a] | 0.41 ± 0.70 [c] | 12.50 ± 5.00 [b] | 5.7e-5 | 0.0004 | 0.0371 |
| 105 | 49.96 ± 4.37 [a] | 0.89 ± 0.78 [c] | 14.17 ± 5.64 [b] | 1.6e-5 | 0.0001 | 0.0182 |
| 120 | 51.66 ± 4.34 [a] | 0.89 ± 0.78 [c] | 14.17 ± 5.64 [b] | 1.3e-5 | 7.8e-5 | 0.0180 |
| 135 | 53.90 ± 5.28 [a] | 1.30 ± 1.23 [c] | 15.00 ± 5.00 [b] | 1.3e-5 | 7.5e-5 | 0.0179 |
| 150 | 55.52 ± 5.54 [a] | 1.30 ± 1.23 [c] | 15.83 ± 5.20 [b] | 1.4e-5 | 8.6e-5 | 0.0167 |
| 165 | 56.60 ± 6.27 [a] | 1.78 ± 1.56 [c] | 16.25 ± 4.51 [b] | 1.5e-5 | 8.9e-5 | 0.0188 |
| 180 | 58.21 ± 7.06 [a] | 1.78 ± 1.56 [c] | 16.67 ± 3.82 [b] | 1.5e-5 | 9.3e-5 | 0.0195 |
| 195 | 58.77 ± 7.88 [a] | 2.93 ± 0.72 [c] | 17.08 ± 4.02 [b] | 2.7e-5 | 0.0001 | 0.0341 |
| 210 | 58.77 ± 7.88 [a] | 3.41 ± 1.09 [c] | 17.92 ± 3.61 [b] | 2.6e-5 | 0.0001 | 0.0289 |
| 225 | 59.32 ± 8.73 [a] | 3.41 ± 1.09 [c] | 17.92 ± 3.61 [b] | 4.0e-5 | 0.0002 | 0.0406 |
| 240 | 59.32 ± 8.73 [a] | 3.41 ± 1.09 [c] | 18.33 ± 2.89 [b] | 3.4e-5 | 0.0002 | 0.0326 |

One-way analysis of variance (ANOVA) along with Fisher's least significant difference tests was used. Data are presented as the mean ± standard deviation (mean ± SD) and differences between treatments are considered significant when $P < 0.05$. All statistical analyses were conducted using SPSS 26.0 Statistics and EXCEL.

**Supplementary Table 5 Detailed statistics analysis of Fig. 4c (*D. yakuba*)**

| Time (min) | $P_{Lb}$ | CK | $P_{dsEsGAP1}$ | *P* value | | |
|---|---|---|---|---|---|---|
| | | | | $P_{Lb}$ vs CK | $P_{Lb}$ vs $P_{dsEsGAP1}$ | CK vs $P_{dsEsGAP1}$ |
| 0 | 0.00 ± 0.00 | 0.00 ± 0.00 | 0.00 ± 0.00 | — | — | — |
| 15 | 2.24 ± 1.94 $^{a}$ | 0.00 ± 0.00 $^{a}$ | 0.00 ± 0.00 $^{a}$ | 0.1096 | 0.1096 | 0.9999 |
| 30 | 6.38 ± 2.98 $^{a}$ | 0.00 ± 0.00 $^{b}$ | 0.33 ± 0.58 $^{b}$ | 0.0102 | 0.0131 | 0.9707 |
| 45 | 14.22 ± 2.60 $^{a}$ | 0.00 ± 0.00 $^{b}$ | 2.33 ± 1.15 $^{b}$ | 0.0001 | 0.0003 | 0.2668 |
| 60 | 20.59 ± 2.75 $^{a}$ | 0.24 ± 0.41 $^{c}$ | 5.00 ± 1.73 $^{b}$ | 2.9e-5 | 0.0001 | 0.0491 |
| 75 | 26.24 ± 6.30 $^{a}$ | 0.24 ± 0.41 $^{b}$ | 5.33 ± 2.08 $^{b}$ | 0.0004 | 0.0013 | 0.3060 |
| 90 | 29.82 ± 4.75 $^{a}$ | 0.47 ± 0.82 $^{b}$ | 5.67 ± 2.52 $^{b}$ | 6.6e-5 | 0.0002 | 0.1869 |
| 105 | 37.35 ± 5.93 $^{a}$ | 1.13 ± 1.07 $^{b}$ | 8.00 ± 1.73 $^{b}$ | 4.4e-5 | 0.0001 | 0.1276 |
| 120 | 41.76 ± 6.29 $^{a}$ | 1.13 ± 1.07 $^{c}$ | 9.00 ± 2.00 $^{b}$ | 3.3e-5 | 0.0001 | 0.1023 |
| 135 | 44.06 ± 6.87 $^{a}$ | 1.13 ± 1.07 $^{c}$ | 10.67 ± 2.52 $^{b}$ | 4.3e-5 | 0.0002 | 0.0755 |
| 150 | 47.55 ± 6.38 $^{a}$ | 1.53 ± 0.52 $^{c}$ | 11.33 ± 2.89 $^{b}$ | 2.1e-5 | 8.5e-5 | 0.0569 |
| 165 | 50.56 ± 5.64 $^{a}$ | 1.53 ± 0.52 $^{c}$ | 11.67 ± 3.21 $^{b}$ | 9.0e-6 | 3.7e-5 | 0.0377 |
| 180 | 53.33 ± 4.79 $^{a}$ | 1.53 ± 0.52 $^{c}$ | 12.00 ± 3.46 $^{b}$ | 3.6e-6 | 1.5e-5 | 0.0224 |
| 195 | 55.62 ± 3.79 $^{a}$ | 1.94 ± 0.67 $^{c}$ | 12.00 ± 3.46 $^{b}$ | 1.5e-6 | 4.5e-6 | 0.0146 |
| 210 | 56.22 ± 4.01 $^{a}$ | 2.35 ± 0.19 $^{c}$ | 12.33 ± 3.79 $^{b}$ | 1.9e-6 | 6.4e-6 | 0.0201 |
| 225 | 59.59 ± 1.91 $^{a}$ | 2.35 ± 0.19 $^{c}$ | 12.33 ± 3.79 $^{b}$ | 4.8e-7 | 1.1e-6 | 0.0059 |
| 240 | 59.59 ± 1.91 $^{a}$ | 2.35 ± 0.19 $^{c}$ | 12.67 ± 4.16 $^{b}$ | 6.7e-7 | 1.6e-6 | 0.0074 |

One-way analysis of variance (ANOVA) along with Fisher's least significant difference tests was used. Data are presented as the mean ± standard deviation (mean ± SD) and differences between treatments are considered significant when $P < 0.05$. All statistical analyses were conducted using SPSS 26.0 Statistics and EXCEL.
