# Supplementary material for: Neofunctionalization of an ancient domain allows parasites to avoid intraspecific competition by manipulating host behaviour
Source: Nat Commun. 2021 Sep 16;12:5489. doi: 10.1038/s41467-021-25727-9 (PMC8446075; doi:10.1038/s41467-021-25727-9)
Supplement: Supplementary file 3 — Description of Additional Supplementary Files [file 41467_2021_25727_MOESM3_ESM.docx]

**Description of Additional Supplementary Files**

**Supplementary Data 1: Description**

The information of primers used in this study.

**Supplementary Movie 1: Description**

No escape behaviors of Drosophila melanogaster 2nd instar larvae without exposure to parasitoids.

**Supplementary Movie 2: Description**

Escape behaviors of Drosophila melanogaster 2nd instar larvae upon the parasitization of Leptopilina boulardi.
