# Supplementary material for: Neofunctionalization of an ancient domain allows parasites to avoid intraspecific competition by manipulating host behaviour
Source: Nat Commun. 2021 Sep 16;12:5489. doi: 10.1038/s41467-021-25727-9 (PMC8446075; doi:10.1038/s41467-021-25727-9)
Supplement: Supplementary file 7 — Reporting Summary [file 41467_2021_25727_MOESM7_ESM.pdf]

nature research

Corresponding author(s): Xuexin Chen; Shuai Zhan; Jianhua Huang

Last updated by author(s): Aug 8, 2021

Reporting Summary

Nature Research wishes to improve the reproducibility of the work that we publish. This form provides structure for consistency and transparency in reporting. For further information on Nature Research policies, see our Editorial Policies and the Editorial Policy Checklist.

Statistics

For all statistical analyses, confirm that the following items are present in the figure legend, table legend, main text, or Methods section.

n/a	Confirmed	
☐	☒	The exact sample size (*n*) for each experimental group/condition, given as a discrete number and unit of measurement
☐	☒	A statement on whether measurements were taken from distinct samples or whether the same sample was measured repeatedly
☐	☒	The statistical test(s) used AND whether they are one- or two-sided<br>*Only common tests should be described solely by name; describe more complex techniques in the Methods section.*
☒	☐	A description of all covariates tested
☐	☒	A description of any assumptions or corrections, such as tests of normality and adjustment for multiple comparisons
☐	☒	A full description of the statistical parameters including central tendency (e.g. means) or other basic estimates (e.g. regression coefficient) AND variation (e.g. standard deviation) or associated estimates of uncertainty (e.g. confidence intervals)
☐	☒	For null hypothesis testing, the test statistic (e.g. F, t, r) with confidence intervals, effect sizes, degrees of freedom and P value noted<br>*Give P values as exact values whenever suitable.*
☒	☐	For Bayesian analysis, information on the choice of priors and Markov chain Monte Carlo settings
☒	☐	For hierarchical and complex designs, identification of the appropriate level for tests and full reporting of outcomes
☒	☐	Estimates of effect sizes (e.g. Cohen's *d*, Pearson's *r*), indicating how they were calculated

Our web collection on statistics for biologists contains articles on many of the points above.

Software and code

Policy information about availability of computer code

Data collection	Zeiss Zen was used to collect the confocal images.
Data analysis	Image analysis: ImageJ v1.52j;<br>Kmer analysis: Jellyfish v2.2.3;<br>Genome assembly (long reads): Canu v2.0 (removing errors), Flye v2.7-b1585 (assembling), pilon v1.2.3 (polish);<br>Assembly assessment: CEGMA v2.4, BUSCO v3;<br>Repeat annotation: RepeatModeler v1.0.7 (generating species-specific library), RepeatMasker v4.0.5;<br>Gene prediction: Marker v2.31.10 (combining signatures), AUGUSTUS v3.3.2 (providing ab initio signatures), SNAP v2006-07-28 (providing ab initio signatures), cDNA_Cupcake v6.4 (processing PacBio RNAseq data), StringTie v2.0 (processing illumina RNAseq data);<br>Gene annotation: BLASTP (alignming genes to other gene sets or databases), InterProScan v5.13 (mapping genes with functional terms);<br>Gene prediction of RhoGAP genes in Leptopilina: TBLASTN (mapping homologs to the genome), GeneWise v2.2.26 (predicting genes based on homology);<br>Multiple alignment: MultAlin (online);<br>Phylogenetic analysis: RAxML v8.2.10;<br>Expression profiling: Salmon v0.12.0;<br>Heatmap and clustering: pheatmap of R v4.0.0.

For manuscripts utilizing custom algorithms or software that are central to the research but not yet described in published literature, software must be made available to editors and reviewers. We strongly encourage code deposition in a community repository (e.g. GitHub). See the Nature Research guidelines for submitting code & software for further information.

Data

Policy information about availability of data

All manuscripts must include a data availability statement. This statement should provide the following information, where applicable:

- Accession codes, unique identifiers, or web links for publicly available datasets
- A list of figures that have associated raw data
- A description of any restrictions on data availability

The transcript sequences of three Lb EsGAP genes used in this study have been deposited in GenBank with the accession numbers MZ673645 (EsGAP1), MZ673646 (EsGAP2) and MZ673647 (EsGAP3). The genome assembly generated in this study has been deposited in GenBank BioProject under accession number PRJNA671782. The proteome data of the Lb venom fluids has been deposited in PeptideAtlas under the accession number PASS01481. Source data are provided with this paper.

Field-specific reporting

Please select the one below that is the best fit for your research. If you are not sure, read the appropriate sections before making your selection.

☒ Life sciences ☐ Behavioural & social sciences ☐ Ecological, evolutionary & environmental sciences

For a reference copy of the document with all sections, see nature.com/documents/nr-reporting-summary-flat.pdf

Life sciences study design

All studies must disclose on these points even when the disclosure is negative.

Sample size	Sample size was predetermined based on previous studies in this field (at least 100 animals for behavioral experiments and at least 10 animals for confocal experiments). These numbers of samples were sufficient to perform a confident data analysis. All sample sizes are provided in each figure legend and source data.
Data exclusions	No data was excluded from our analyses.
Replication	All data presented are representative of at least three independent experiments as indicated in the figure legends, and replications were successful.
Randomization	In all experiment wasps or flies were randomly assigned to experimental groups. Specifically, the samples for DNA sequencing, transcriptome sequencing, LC-MS, western blot and qRT-PCR were all randomly selected. The host larvae were also randomly picked to test the escape behavior and the wasp superparasitism behavior. The central nervous system (CNS) were dissected from a certain number of randomly-selected parasitized host larvae for detect the intracellular ROS levels and antibody staining.
Blinding	Investigators were blinded to group allocation of dsRNA-treated Lb females for detecting the host escape index. Blinding was not required for other experiments, because sample preparation, data collection and image analysis were performed using the same conditions for all the samples regardless of their identity.

Reporting for specific materials, systems and methods

We require information from authors about some types of materials, experimental systems and methods used in many studies. Here, indicate whether each material, system or method listed is relevant to your study. If you are not sure if a list item applies to your research, read the appropriate section before selecting a response.

Materials & experimental systems

n/a	Involved in the study	
☐	☒	Antibodies
☒	☐	Eukaryotic cell lines
☒	☐	Palaeontology and archaeology
☐	☒	Animals and other organisms
☒	☐	Human research participants
☒	☐	Clinical data
☒	☐	Dual use research of concern

Methods

n/a	Involved in the study	
☒	☐	ChIP-seq
☒	☐	Flow cytometry
☒	☐	MRI-based neuroimaging

Antibodies

Antibodies used	anti-EsGAP1 (ABclonal, rabbit polyclonal, 1:500)<br>anti rabbit IgG, HRP (Solarbio, Cat#SE134, from goat serum, 1:2000)<br>Alexa Fluor 594 Goat anti-Rabbit second antibody (Molecular Probes, Cat#A11012, 1:1000)

Validation	anti-EsGAP1: generated by ABclonal and Western Blot and Immunostaining reactivity validated in this study<br>anti rabbit IgG, HRP: statement from Solarbio, validated for Western Blot<br>Alexa Fluor 594 Goat anti-Rabbit second antibody: Immunostaining-Published species (Thermofisher Sci website, under product specification)

Animals and other organisms

Policy information about studies involving animals; ARRIVE guidelines recommended for reporting animal research

Laboratory animals	Only invertebrate species (Drosophila flies and parasitoid wasps) are used in this study. Information of strains and species is provided in the Methods under the section "Insects".
Wild animals	N/A
Field-collected samples	N/A
Ethics oversight	N/A

Note that full information on the approval of the study protocol must also be provided in the manuscript.
